# Clinical reference values for left ventricular function and synchronicity parameters evaluated by GMPI in patients with a low pretest likelihood of coronary artery disease

**DOI:** 10.3389/fcvm.2026.1696511

**Published:** 2026-03-27

**Authors:** Yufeng Wang, Lingling Wang, Lei Li, Xiaohui Ouyang, Xiaohong Zhou, Xiaofei Liu

**Affiliations:** 1 Department of Nuclear Medicine, The Eighth Medical Center of the Chinese People’s Liberation Army General Hospital, Beijing, China; 2 Department of Ultrasound Medicine, The Eighth Medical Center of the Chinese People’s Liberation Army General Hospital, Beijing, China

**Keywords:** clinical reference values, GMPI, left ventricular function parameters, left ventricular synchronicity parameters, left ventricular volume

## Abstract

**Background:**

Left ventricular function and synchronicity parameters are significant for the diagnosis, risk stratification, and prognosis evaluation of cardiovascular diseases. The purpose of this study was to determine the clinical reference values for left ventricular function and synchronicity parameters evaluated by gated myocardial perfusion imaging (GMPI) using three quantitative software packages.

**Methods:**

A cohort of 142 consecutive patients with low-risk coronary artery disease who underwent stress gated myocardial perfusion imaging was retrospectively collected. In our center, a portion of subjects were derived from the cadre department, who regularly participate in physical activity. The population was further grouped based on different physical activity levels, i.e., individuals who participated in regular physical activity vs. those who were sedentary. Data on left ventricular function and synchronicity parameters were collected by three quantitative software packages: Corridor 4-dimensional model (4D-M), quantitative gated single-photon emission computed tomography (QGS), and Emory Cardiac Toolbox (ECTb). The left ventricular function parameters included ejection fraction (EF), end-diastolic volume (EDV), and end-systolic volume (ESV). EDV and ESV were corrected for body surface area (BSA) as end-diastolic volume index (EDVI) and end-systolic volume index (ESVI). The clinical reference ranges for left ventricular function and synchronicity parameters in patients with low-risk coronary artery disease were based on 95% CIs.

**Results:**

There were significant differences in the left ventricular function (EF, EDV, and EDVI) and left ventricular synchronicity parameters obtained by the three quantitative software packages. There were no significant differences in ESV and ESVI. The multiple linear regression analysis showed that gender, age, and BMI were important variables for EDV and ESV. Age and gender were found to be the significant variables for EDVI, ESVI, and EF. For the men, the lower reference range for EF calculated by the three algorithms was 58%, 54%, and 58%, respectively; the upper range for EDV was 135, 116, and 131 mL, respectively; and the upper range of ESV was 51, 49, and 52 mL, respectively. For the women, the lower reference range for EF was 71%, 61%, and 65%, respectively; the upper range of EDV was 99, 86, and 96 mL, respectively; and the upper range for ESV was 28, 28, and 30 mL, respectively. The upper range for BW was 29.7${}^{\circ }$, 53.1${}^{\circ }$, and 50.9${}^{\circ }$, respectively. The upper range for SD was 7.9${}^{\circ }$, 17.8${}^{\circ },$ and 19.0${}^{\circ }$, respectively. The upper range for E obtained by QGS was 47${}^{\circ }$. Compared to the women, the men had larger left ventricular volume values, lower mean EF values, and a lower prevalence of having a small heart. There were no differences in left ventricular synchronicity parameters between the men and women. After 1:1 propensity score matching for age and BMI, 37 men and 37 women were matched. The results were consistent before and after propensity score matching.

**Conclusion:**

This study determined the clinical reference values for left ventricular function and synchronicity parameters evaluated by GMPI using quantitative software. Compared to the women, EDV, ESV, EDVI, and ESVI in the men were higher, while EF and the prevalence of having a small heart were lower. There were no differences in left ventricular synchronicity parameters between the men and women.

## Introduction

Left ventricular ejection fraction (LVEF) and left ventricular volumes are important parameters for the diagnosis, risk stratification, and prognosis evaluation of patients with coronary heart disease (CHD) (1, 2). Left ventricular dyssynchrony is associated with left ventricular remodeling and adverse cardiac events (3). Gated myocardial perfusion imaging (GMPI), as a non-invasive imaging modality for evaluating myocardial ischemia and left ventricular function simultaneously, can accurately obtain critical left ventricular functional and synchronicity parameters. Quantitative analysis is the critical strength of GMPI, which can obtain left ventricular function parameters such as LVEF, left ventricular volumes, and left ventricular synchronicity parameters using GMPI quantitative software. Compared to other imaging modalities, GMPI is convenient, reproducible, and automatic.

However, the normal reference values for existing left ventricular function parameters are predominantly based on data from European and American populations (4) and exhibit significant racial and regional differences. Nakajima et al. (5) noted that Japanese populations exhibit significantly smaller left ventricular volumes than Western cohorts. Mukherjee et al. (6) have also found that cardiac mechanical synchrony parameters in the Indian population significantly differ from the Western reference values, suggesting that geographical and racial factors may further interfere with diagnostic thresholds. Previous studies have demonstrated that gender (7), body weight (5), imaging instrumentation, and reconstruction methods (8) significantly influence these values. Common GMPI quantitative software includes Cedars-Sinai quantitative gated single-photon emission computed tomography (QGS) (9), the Corridor 4-dimensional model (4DM) (10), and the Emory Cardiac Toolbox (ECTb, Emory University, USA) (11). There are differences in left ventricular function parameters obtained by different software (12–14).

Previous studies have established normal reference values for left ventricular function parameters in Chinese populations. Li et al. (12) established the reference limits for cardiac parameters calculated by three quantitative software packages based on 175 patients with low-risk coronary artery disease. Wang et al. (15) established reference ranges for resting GMPI left ventricular synchronicity, obtained using the Cedars-Sinai QGS software, in 74 healthy subjects. Meng et al. (16) established normal reference values for stress GMPI left ventricular function parameters in low-probability patients with stable coronary artery disease (SCAD), acquired using cardiac cadmium-zinc-telluride (CZT) single-photon emission computed tomography (SPECT).

However, there are differences among different centers, including the equipment used, collection conditions, and reconstruction methods. In addition, ultrasound data were not included in previous studies. In our population, a portion of the subjects were derived from the cadre department, who regularly participate in physical activity. This may differ from the data collected by other centers. Therefore, we aim to develop clinical reference ranges for these parameters based on the available data from our center. The purpose of this study was to establish clinical reference ranges for left ventricular function and synchronicity parameters in GMPI using three quantitative software packages based on subjects with a low pretest likelihood of coronary artery disease at our center. These ranges are clinically important for improving the diagnostic accuracy of coronary heart disease and guiding individualized treatment.

## Methods

### Study population

This was a single-center study that retrospectively enrolled subjects who underwent stress gated myocardial perfusion imaging at the Eighth Medical Center of the Chinese People's Liberation Army General Hospital from September 2023 to February 2025. Inclusion criteria were as follows: (1) subjects without a known history of cardiac disease (e.g., CAD, cardiomyopathy, heart valve disease, and severe arrhythmia); (2) subjects with a low-pretest likelihood of coronary artery disease. Low-pretest likelihood of coronary artery disease (<30%) was defined according to the updated Diamond–Forrester criteria (17). Data on age, sex, and type of chest pain were collected. Chest pain was classified into the following three categories: typical, atypical, and non-specific. Typical chest pain was characterized by (I) substernal discomfort, (II) provocation by physical exertion or emotional stress, and (III) relief upon rest or administration of nitroglycerin. Atypical chest pain met two of the aforementioned criteria. Patients exhibiting one or none of these criteria were classified as having non-specific chest pain; (3) subjects with normal perfusion, regional wall motion at stress GMPI; (4) normal ECG at rest and during stress test; (5) EF >50% on GMPI quantitative software. Clinical data, including height, weight, hypertension, diabetes, and hyperlipemia, were collected. BMI was calculated by height and weight. Body surface area (BSA) was calculated using a modified Stevenson formula for Chinese populations: BSA = 0.0061 ×height (cm) + 0.0124 × weight (kg) − 0.0099. The population was further grouped based on different physical activity levels, i.e., individuals who participated in regular physical activity vs. those who were sedentary. The study was approved by the Ethics Committee of the Eighth Medical Center of the Chinese People's Liberation Army General Hospital.

### Echocardiography acquisition

In total, 128 individuals underwent M-model echocardiography (Vivid E95, GE Medical Systems, USA). Ejection fraction (EF), end-diastolic volume (EDV), and end-systolic volume (ESV) were acquired using M-mode echocardiography.

### GMPI acquisition

Exercise and pharmaceutical stress gated myocardial perfusion imaging were performed 60–90 min after injection of ^99m^Tc-MIBI (740 MBq, radiochemical purity > 95%, Beijing Atom High-Tech Co., Ltd., Beijing, China) using a two-detector 90${}^{\circ }$ camera (Optima NM/CT 640, GE Medical Systems, USA). One hundred eighteen subjects performed pharmaceutical stress GMPI while 24 subjects performed exercise treadmill stress GMPI. The images were acquired with a low-energy and high-resolution parallel-hole collimator, photopeak with 140-keV and 20% symmetric energy window, matrix 64 × 64 and magnification 1.6. ECG-gated images were performed with eight frames per cardiac, cycle 3${}^{\circ }$/view and 20s/view. CT attenuation correction was not utilized for gated SPECT-MPI images.

### GMPI processing

The raw images were reconstructed using the filtered back projection method (filter cutoff: 0.4, filter power: 10) without CT attenuation correction. MPI images were evaluated by consensus of two experienced observers, who were blind to the patients' clinical data. The final decision was made by the senior physician in case of disagreement. Myocardial perfusion was divided into 17-segment using a five-point score system (0: normal to 4: absence of tracer uptake) (18). The summed stress score (SSS) was calculated automatically by software QPS (version 2009). Normal stress myocardial perfusion was assessed by visual and quantitative scoring (SSS<4) (19). Wall motion abnormality was also confirmed by the two physicians. Left ventricular function parameters including EF, EDV and ESV were obtained. EDV and ESV were corrected by BSA to obtain end-diastolic volume index (EDVI) and end-systolic volume index (ESVI) respectively. Left ventricular synchronicity parameters included phase bandwidth (PBW) and phase standard deviation (PSD). All the parameters were obtained by using stress GMPI. All the analyses were performed using standard quantitative software settings in Corridor 4DM, QGS, and ECTb. The software versions used in this study were version v2017 for Corridor 4DM, version 2009 for QGS, and version 4.2 for Emory Cardiac Toolbox.

### Statistical analysis

IBM SPSS (Version 26.0, SPSS Inc., Chicago, IL, USA) was used for the statistical analysis in this study. Continuous data are expressed as mean ± SD when distributed normally and median (Q1–Q3) when distributed non-normally. Categorial variables are expressed as percentages. The Shapiro–Wilk test was used to assess the normality of each variable. The reference thresholds were obtained using normal distribution or percentiles. The 95th percentiles of the ranges were calculated using the formula for normal distribution. EF, EDV, EDVI, ESV, ESVI, BW, SD, and entropy (E) were one-sided cutoffs. An EF value that was too low was considered abnormal. A lower EF limit was required. EDV, EDVI, ESV, ESVI, BW, and SD values that were too high were considered abnormal. The upper limits of these parameters were required. In order to evaluate the influence of age, gender, BMI, and physical activity on EF, EDV, ESV, EDVI, and ESVI, a multiple linear regression analysis was conducted. The presence of collinearity was ascertained through the evaluation of the variance inflation factor (VIF). Problematic collinearity was defined as VIF >5. The chi-square test was used for categorical variables. Propensity score matching based on the ages of the female and male participants (1:1 ratio) was used to adjust for potential confounding factors. *P* < 0.05 was considered statistically significant.

## Results

### Baseline characteristics

This study included 142 subjects (105 men and 37 women, 45 ± 17 years old, 24.7 ± 3.6 kg/m^2^). The baseline characteristics are shown in Table 1. In total, 14 subjects had diabetes, 44 subjects had hypertension, and 48 subjects had hyperlipidemia. Furthermore, 118 subjects underwent pharmaceutical stress GMPI, while 24 subjects underwent exercise treadmill stress GMPI.

**Table 1:** Baseline characteristics of the study population.

Clinical data	Subjects (*n* = 142)
Male, *n* (%)	105 (74)
Age (year)	45 ± 17
BMI (kg/m^2^)	24.7 ± 3.6
BSA (m^2^)	1.9 ± 0.2
Diabetes, *n* (%)	14 (10)
Hypertension, *n* (%)	44 (31)
Hyperlipidemia, *n* (%)	48 (34)
Pharmaceutical stress, *n* (%)	118 (83)
Exercise treadmill stress, *n* (%)	24 (17)

Data are shown as mean (SD) for continuous variables or as a percentage for categorical variables.

BMI, body mass index; BSA, body surface area.

### Comparison of three quantitative software packages in evaluating left ventricular function and synchronicity parameters

**Figure 1:**
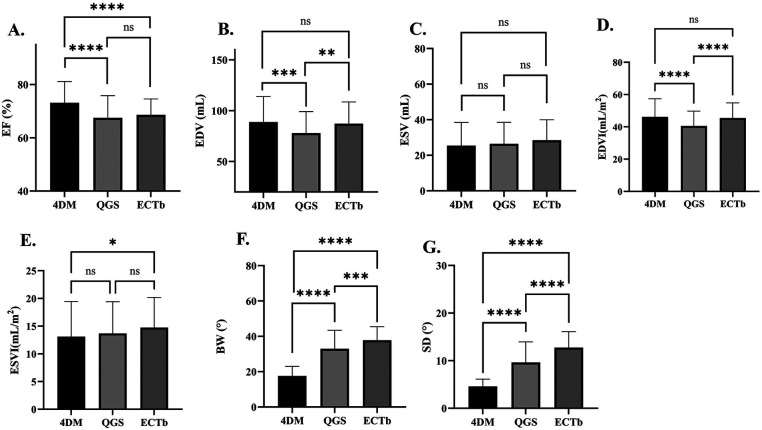
Pairwise comparisons of EF **(A)**, EDV **(B)**, ESV **(C)**, EDVI **(D)**, ESVI **(E)**, BW **(F)**, and SD **(G)** values derived using 4DM, QGS, and ECTb. 4DM, Corridor 4-dimensional model; ECTb, Emory Cardiac Toolbox; QGS, quantitative gated single-photon emission computed tomography; EF, ejection fraction; EDV, end-diastolic volume; EDVI, end-diastolic volume index; ESV, end-systolic volume; ESVI, end-systolic volume index; BW, bandwidth; SD, standard deviation.

The values for the left ventricular function and synchronicity parameters are presented in Table 2. The mean EF was 73 ± 8.0%, 67 ± 8.3%, and 69 ± 6.0% for 4DM, QGS, and ECTb, respectively. The mean EDV was 89 ± 25.0, 78 ± 21.5, and 87 ± 21.4 mL, respectively. The mean ESV was 25 ± 13.0, 27 ± 12.0, and 28 ± 11.5 mL, respectively. The mean BW was 18 ± 5.5${}^{\circ }$, 33 ± 10.8${}^{\circ }$, and 38 ± 7.7${}^{\circ }$, respectively. The mean SD was 4.6 ± 1.5${}^{\circ }$, 9.7 ± 4.3${}^{\circ }$, and 12.8 ± 3.3${}^{\circ }$, respectively. A comparison of the three quantitative software packages in evaluating left ventricular function and synchronicity parameters is shown in Figure 1. EF was comparable between QGS and ECTb (*P* = n.s.), both of which were lower than that of 4DM (*P* < 0.001). EDV and EDVI were comparable between 4DM and ECTb (*P* = n.s.), both of which were higher than those of QGS (*P* < 0.05). ESV was comparable between 4DM, QGS, and ECTb (*P* = n.s.). ESVI obtained using 4DM was lower than that of ECTb (*P* < 0.05). BW and SD were the highest when using ECTb, followed by QGS and 4DM (*P* < 0.001).

**Table 2:** Comparison of the three quantitative software packages in the evaluation of left ventricular function and synchronicity parameters.

Quantitative software package	EF (%)	EDV (mL)	EDVI (mL/m^2^)	ESV (mL)	ESVI (mL/m^2^)	BW (${}^{\circ }$)	SD (${}^{\circ }$)	E (${}^{\circ }$)
GMPI								
4DM	73 ± 8.0	89 ± 25.0	46 ± 11.2	25 ± 13.0	13 ± 6.3	18 ± 5.5	4.6 ± 1.5	
QGS	67 ± 8.3	78 ± 21.5	40 ± 9.3	27 ± 12.0	14 ± 5.7	33 ± 10.8	9.7 ± 4.3	35.7 ± 7.4
ECTb	69 ± 6.0	87 ± 21.4	45 ± 9.4	28 ± 11.5	15 ± 5.4	38 ± 7.7	12.8 ± 3.3	
*P*-value	<0.001	<0.001	<0.001	0.104	0.056	<0.001	<0.001	

Data are shown as mean (SD).

EF, ejection fraction; EDV, end-diastolic volume; EDVI, end-diastolic volume index; ESV, end-systolic volume; ESVI, end-systolic volume index; BW, bandwidth; SD, standard deviation; E, entropy; 4DM, Corridor 4-dimensional model; QGS, quantitative gated single-photon emission computed tomography; ECTb, Emory Cardiac Toolbox.

### Comparison of left ventricular function parameters obtained by GMPI and echocardiography

The means ± SD of EF, EDV, and ESV, measured using echocardiography or the three software packages, are shown in Table 3. In total, 128 subjects were measured using two-dimensional echocardiography. There were significant differences in the EF and ESV values between those measured using echocardiography and the three software packages; however, EDV measured using echocardiography, 4DM, and ECTb showed no significant differences. The EDV values derived using QGS were significantly lower than those measured using echocardiography.

**Table 3:** Comparison of EF, EDV, and ESV values derived from 4DM, QGS, ECTb, and echocardiography.

Parameters	Echocardiography (*n* = 128)	4DM	QGS	ECTb	*P*-value*	*P-*value**	*P*-value***
EF (%)	62 ± 1.9	73 ± 8.0	67 ± 8.3	69 ± 6.0	<0.001	<0.001	<0.001
EDV (mL)	92 ± 17.3	89 ± 25.0	78 ± 21.5	87 ± 21.4	0.442	<0.001	0.128
ESV (mL)	35 ± 7.5	25 ± 13.0	27 ± 12.0	28 ± 11.5	<0.001	<0.001	<0.001

Data are shown as mean (SD).

EF, ejection fraction; EDV, end-diastolic volume; ESV, end-systolic volume; 4DM, Corridor 4-dimensional model; QGS, quantitative gated single-photon emission computed tomography; ECTb, Emory Cardiac Toolbox.

* Comparison between echocardiography and 4DM.

** Comparison between echocardiography and QGS.

*** Comparison between echocardiography and ECTb.

### Comparison of left ventricular function and synchronicity parameters obtained by three quantitative software in men and women

A comparison of left ventricular function parameters obtained using three quantitative software in men and women is shown in Table 4. The male subjects had higher BMI, EDV, and ESV, while the female subjects had significantly higher age and EF. Having a small heart was defined as an ESV less than 20 mL when measured using QGS (20). A lower proportion of the men had a small heart than among the women (4DM: 23% vs. 73%, QGS: 19% vs. 73%, ECTb: 13% vs. 59%, all *P* < 0.001). There were no significant differences in the BW and SD values obtained using the three algorithms between the men and women.

**Table 4:** Comparison of the left ventricular function and synchronicity parameters obtained by different software packages between the sexes.

Parameters	Male subjects (*n* = 105)	Female subjects (*n* = 37)	*P*-value
Age (years)	45 ± 16.7	58 ± 15.2	<0.001
BMI (kg/m^2^)	24.9 ± 3.5	24.3 ± 3.9	0.43
BSA (m^2^)	1.96 (1.86, 2.06)	1.70 (1.64, 1.80)	<0.001
Diabetes, *n* (%)	13 (12)	5 (14)	0.86
Hypertension, *n* (%)	32 (30)	12 (32)	0.83
Hyperlipidemia, *n* (%)	34 (32)	14 (38)	0.55
Pharmaceutical stress, *n* (%)	87 (83)	31 (84)	0.90
Exercise treadmill stress, *n* (%)	18 (17)	6 (16	0.90
4DM			
EF (%)	71 ± 7.3	79 ± 6.7	<0.001
EDV (mL)	97 ± 22.3	66 ± 17.9	<0.001
ESV (mL)	29 ± 12.4	15 ± 7.3	<0.001
EDVI (mL/m^2^)	49 ± 10.4	37.9 ± 9.3	<0.001
ESVI (mL/m^2^)	14.8 ± 6.1	8.3 ± 4.1	<0.001
BW (${}^{\circ }$)	18 ± 5.6	17 ± 5.4	0.47
SD (${}^{\circ }$)	4.6 ± 1.5	4.6 ± 1.6	0.75
QGS			
EF (%)	65 ± 7.3	74 ± 7.6	<0.001
EDV (mL)	85 ± 18.9	58 ± 14.0	<0.001
ESV (mL)	30 ± 11.0	16 ± 7.2	<0.001
EDVI (mL/m^2^)	43.0 ± 8.6	33.3 ± 7.5	<0.001
ESVI (mL/m^2^)	15.3 ± 5.3	9.0 ± 4.0	<0.001
Small heart (%)	20/105 (19)	27/37 (73)	<0.001
BW (${}^{\circ }$)	34 ± 10.0	30 ± 12.7	0.07
SD (${}^{\circ }$)	10.1 ± 4.1	8.4 ± 4.7	0.07
E (%)	37 ± 6.6	33 ± 8.5	0.003
ECTb			
EF (%)	67 ± 5.5	73 ± 5.1	<0.001
EDV (mL)	94 ± 18.9	67 ± 15.0	<0.001
ESV (mL)	32 ± 10.9	19 ± 6.4	<0.001
EDVI (mL/m^2^)	47.7 ± 8.6	39.0 ± 8.7	<0.001
ESVI (mL/m^2^)	16.2 ± 5.2	10.7 ± 3.7	<0.001
BW (${}^{\circ }$)	38 ± 6.8	37 ± 9.8	0.35
SD (${}^{\circ }$)	13 ± 3.1	12 ± 3.8	0.19

Data are shown as mean (SD) or median (IQR) for continuous variables, or as percentages for categorical variables.

BMI, body mass index; BSA, body surface area; 4DM, Corridor 4-dimensional model; QGS, quantitative gated single-photon emission computed tomography; EF, ejection fraction; EDV, end-diastolic volume; EDVI, end-diastolic volume index; ESV, end-systolic volume; ESVI, end-systolic volume index; BW, bandwidth; SD, standard deviation; E, entropy; ECTb, Emory Cardiac Toolbox.

### Age-related differences in left ventricular function and synchronicity parameters assessed using QGS

In order to evaluate age-related differences in left ventricular function and synchronicity parameters obtained using QGS, three age subgroups (≤40, 41–59, and ≥60 years) were defined among the men (Table 5). Among the men, EDV, ESV, EDVI, and ESVI differed significantly among the three age groups. EF, BW, SD, and E did not differ significantly among the three subgroups. Among the women, because we were limited by the sample size, two subgroups were defined (≤60 and >60 years). EF differed significantly between the two subgroups. However, EDV, ESV, EDVI, and ESVI did not differ significantly between the two subgroups.

**Table 5:** Age-related differences in left ventricular function and synchronicity parameters obtained using QGS.

Parameters	Age group (years)			*P*-value
	≤40	40–60	≥60	
Male subjects				
No.	42	41	22	
BMI (kg/m^2^)	24 ± 2.7	26 ± 3.3	25 ± 4.4	NS
BSA(m^2^)	1.9 (1.9, 2.0)	2.02 (1.9, 2.1)	1.9 (1.8, 2.0)	0.016
EF (%)	64 ± 6.5	65 ± 7.0	67 ± 9.0	NS
EDV (mL)	93 ± 15.9	84 ± 17.3	70 ± 18.7	<0.001
ESV (mL)	34 ± 10.3	30 ± 10.5	24 ± 10.4	<0.001
EDVI (mL/m^2^)	48 ± 7.6	42 ± 7.3	36 ± 7.4	<0.001
ESVI (mL/m^2^)	18 ± 5.1	15 ± 4.9	12 ± 4.8	<0.001
Small heart (%)	3/42 (7)	8/41 (20)	9/22 (41)	0.006
BW (${}^{\circ }$)	33 ± 8.9	34 ± 9.4	36 ± 12.8	NS
SD (${}^{\circ }$)	9 ± 3.4	10 ± 3.6	11 ± 4.1	NS
E (%)	36 ± 6.3	37 ± 6.0	38 ± 8.3	NS
Female subjects				
No.	16		21	
BMI (kg/m^2^)	26 ± 4.1		22 ± 3.4	0.002
BSA (m^2^)	1.7 (1.6,1.8)		1.7 (1.6,1.8)	NS
EF (%)	71 ± 7.6		76 ± 6.9	0.039
EDV (mL)	60 ± 12.4		56 ± 15.3	NS
ESV (mL)	18 ± 7.2		14 ± 6.8	NS
EDVI (mL/m^2^)	35 ± 6.8		32 ± 7.8	NS
ESVI (mL/m^2^)	10 ± 4.1		8 ± 3.7	NS
Small heart (%)	10/16 (63)		17/21 (81)	0.38
BW (${}^{\circ }$)	29 ± 10.1		31 ± 14.5	NS
SD (${}^{\circ }$)	10.1 ± 5.7		7.2 ± 3.5	NS
E (%)	32 ± 8.0		33 ± 9.1	NS

Data are shown as mean (SD) or median (IQR) for continuous variables, or as percentages for categorical variables.

BMI, body mass index; BSA, body surface area; EF, ejection fraction; EDV, end-diastolic volume; EDVI, end-diastolic volume index; ESV, end-systolic volume; ESVI, end-systolic volume index; BW, bandwidth; SD, standard deviation; E, entropy.

### Comparison of left ventricular function parameters obtained using QGS in subgroups of subjects who regularly participated in physical activity and subjects who were sedentary

A comparison of left ventricular function parameters obtained using QGS in two subgroups, namely, subjects who regularly participated in physical activity and those who were sedentary, is shown in Table 6. Among the men, the subgroup of those who regularly participated in physical activity was younger than the sedentary subgroup. They also had greater EDV, ESV, EDVI, and ESVI values (*P* < 0.05). Among the women, the subgroup of those who regularly participated in physical activity was younger and had a lower EF value than the sedentary subgroup. They also had greater ESV (*P* < 0.05).

**Table 6:** Comparison of left ventricular function and synchronicity parameters obtained using QGS between the subgroups of subjects who regularly participated in physical activity and those who were sedentary.

Parameters	Subjects regularly participated in physical activity	Sedentary subjects	*P-*value
Female subjects			
No.	77	30	
Age (years)	35 ± 12.1	64 ± 9.3	<0.001
BMI (kg/m^2^)	24.4 ± 3.3	25.5 ± 3.8	0.10
BSA (m^2^)	1.97 (1.87, 2.06)	1.96 (1.86, 2.05)	0.62
QGS			
EF (%)	68 ± 5.7	76 ± 7.2	0.012
EDV (mL)	63 ± 12.7	57 ± 14.3	0.32
ESV (mL)	21 ± 6.9	14 ± 6.8	0.034
EDVI (mL/m^2^)	37 ± 6.7	33 ± 7.5	0.17
ESVI (mL/m^2^)	12 ± 3.8	8 ± 3.8	0.02
Small heart (%)	3/7 (43)	24/30 (80)	0.13
BW (${}^{\circ }$)	30 ± 11.0	30 ± 13.2	0.97
SD (${}^{\circ }$)	12 ± 6.2	8 ± 4.1	0.14
E (%)	32 ± 7.6	33 ± 8.8	0.84
Male subjects			
No.	61	44	
Age (years)	36 ± 9.1	59 ± 15.6	<0.001
BMI (kg/m^2^)	24.4 ± 3.3	25.5 ± 3.8	0.10
BSA (m^2^)	1.97 (1.87, 2.06)	1.96 (1.86, 2.05)	0.62
QGS			
EF (%)	64 ± 6.6	67 ± 8.0	0.053
EDV (mL)	90 ± 17.7	78 ± 18.3	<0.001
ESV (mL)	33 ± 10.6	27 ± 10.6	0.003
EDVI (mL/m^2^)	46 ± 8.1	39 ± 7.9	<0.001
ESVI (mL/m^2^)	17 ± 5.1	13 ± 5.0	0.002
Small heart (%)	7/61 (11)	13/44 (30)	0.020
BW (${}^{\circ }$)	33 ± 8.5	35 ± 11.8	0.60
SD (${}^{\circ }$)	10.1 ± 4.1	8.4 ± 4.7	0.07
E (%)	37 ± 6.1	37 ± 7.3	0.893

Data are shown as mean (SD) or median (IQR) for continuous variables, or as percentages for categorical variables.

BMI, body mass index; BSA, body surface area; QGS, quantitative gated single-photon emission computed tomography; EF, ejection fraction; EDV, end-diastolic volume; EDVI, end-diastolic volume index; ESV, end-systolic volume; ESVI, end-systolic volume index; BW, bandwidth; SD, standard deviation; E, entropy.

### Multiple linear regression analysis of EF, volume, and volume index obtained using QGS

In order to evaluate the influence of age, gender, BMI, and physical activity on EF, EDV, ESV, EDVI, and ESVI, a multiple linear regression analysis was performed, as shown in Table 7. The collinearity analysis demonstrated no collinearity among the variables (VIF<5, ranging from 1.072 to 2.262). Gender, age, and BMI were important variables for EDV and ESV. Age and gender were found to be significant variables for EDVI, ESVI, and EF.

**Table 7:** Multiple linear regression analysis of EF, volume, and volume index obtained using QGS.

Parameters	Variable	Estimate of coefficient	*P*-value
EDV	Gender	0.391	<0.001
	Age	−0.425	<0.001
	BMI	0.278	<0.001
	Regular physical activity	0.021	0.816
ESV	Gender	0.389	<0.001
	Age	−0.365	<0.001
	BMI	0.192	0.005
	Regular physical activity	0.047	0.622
EDVI	Gender	0.300	<0.001
	Age	−0.449	<0.001
	BMI	0.066	0.341
	Regular physical activity	0.009	0.926
ESVI	Gender	0.313	<0.001
	Age	−0.344	<0.001
	BMI	0.066	0.350
	Regular physical activity	0.039	0.703
EF	Gender	−0.370	<0.001
	Age	0.222	0.022
	BMI	−0.059	0.430
	Regular physical activity	0.083	0.441

EF, ejection fraction; EDV, end-diastolic volume; EDVI, end-diastolic volume index; ESV, end-systolic volume; ESVI, end-systolic volume index; BW, bandwidth; SD, standard deviation; E, entropy.

### Establishment of clinical reference values for left ventricular function and synchronicity parameters obtained using different software packages in men and women

The clinical reference values for left ventricular function (Table 8) and synchronicity parameters (Table 9) obtained using three software packages were established. For men, the lower reference range for EF calculated by the three algorithms was 58%, 54%, and 58%, respectively; the upper range for EDV was 135, 116, and 131 mL and the upper range for ESV was 51, 49, and 52 mL, respectively. For women, the lower reference range for EF was 71%, 61%, and 65%, respectively; the upper range for EDV was 99, 86, and 96 mL, and the upper range for ESV was 28, 28, and 30 mL, respectively. The upper range for BW was 29.7${}^{\circ }$, 53.1${}^{\circ }$, and 50.9${}^{\circ }$, respectively. The upper range for SD was 7.9${}^{\circ }$, 17.8${}^{\circ }$, and 19.0${}^{\circ }$, respectively. The upper range for E obtained using QGS was 47.

**Table 8:** Clinical reference limits for left ventricular function parameters when using the three software packages.

Parameters	4DM		QGS		ECTb	
	Male subjects	Female subjects	Male subjects	Female subjects	Male subjects	Female subjects
EF (%)	≥58	≥71	≥54	≥61	≥58	≥65
EDV (mL)	≤135	≤99	≤116	≤86	≤131	≤96
ESV (mL)	≤51	≤28	≤49	≤28	≤52	≤30
EDVI (mL/m^2^)	≤69	≤54	≤59	≤49	≤64	≤55
ESVI (mL/m^2^)	≤26	≤14	≤25	≤17	≤26	≤17

EF, ejection fraction; EDV, end-diastolic volume; EDVI: end- diastolic volume index; ESV, end-systolic volume; ESVI: end-systolic volume index; 4DM, Corridor 4-dimensional model; QGS, quantitative gated single-photon emission computed tomography; ECTb, Emory Cardiac Toolbox.

**Table 9:** Clinical reference limits for left ventricular synchronicity parameters when using the three software packages.

Parameters	4DM	QGS	ECTb
BW (${}^{\circ }$)	≤29.7	≤53.1	≤50.9
SD (${}^{\circ }$)	≤7.9	≤17.8	≤19.0
E (%)	–	≤47.0	–

BW, bandwidth; SD, standard deviation; E, entropy; 4DM, Corridor 4-dimensional model; QGS, quantitative gated single-photon emission computed tomography; ECTb, Emory Cardiac Toolbox.

### Comparison of left ventricular function and synchronicity parameters obtained using three quantitative software packages between sexes after propensity score matching

There was an imbalance in the male/female participant ratio in this study. The female subjects had a significantly higher age than the male subjects. To increase the reliability of the results, we performed propensity score matching. After propensity score matching, age and BMI were balanced among the male and female subjects (Table 10). There were significant differences in the EF, EDV, ESV, EDVI, and ESVI values obtained using 4DM, QGS, and ECTb between the male and female subjects. There were no significant differences in BW and SD between the sexes.

**Table 10:** Comparison of left ventricular function and synchronicity parameters between the sexes after propensity score matching.

Parameters	Male subjects (37)	Female subjects (37)	*P*-value
Age (year)	59 ± 14.6	58 ± 15.2	0.94
BMI (kg/m^2^)	25.2 ± 3.5	24.3 ± 3.9	0.29
BSA (m^2^)	1.94 (1.85, 2.03)	1.70 (1.65, 1.80)	<0.001
4DM			
EF (%)	72 ± 7.5	79 ± 6.7	<0.001
EDV (mL)	87 ± 18.7	66 ± 17.8	<0.001
ESV (mL)	25 ± 10.2	14 ± 7.3	<0.001
EDVI (mL/m^2^)	45 ± 8.8	38 ± 9.3	0.002
ESVI (mL/m^2^)	13 ± 5.2	8 ± 4.1	<0.001
BW (${}^{\circ }$)	17.8 ± 6.6	17.3 ± 5.4	0.73
SD (${}^{\circ }$)	4.5 ± 1.7	4.6 ± 1.6	0.92
QGS			
EF (%)	67 ± 8.1	74 ± 7.6	<0.001
EDV (mL)	78 ± 16.5	58 ± 14.0	<0.001
ESV (mL)	26 ± 9.3	16 ± 7.2	<0.001
EDVI (mL/m^2^)	40 ± 7.0	33 ± 7.5	<0.001
ESVI (mL/m^2^)	13 ± 4.5	9 ± 4.0	<0.001
BW (${}^{\circ }$)	35 ± 10.7	30 ± 12.7	0.10
SD (${}^{\circ }$)	8.9 ± 3.3	8.4 ± 4.7	0.65
E (%)	38 ± 7.6	33 ± 8.5	0.008
ECTb			
EF (%)	68 ± 5.8	73 ± 5.1	<0.001
EDV (mL)	87 ± 18.0	67 ± 14.9	<0.001
ESV (mL)	28 ± 9.1	18.5 ± 6.4	<0.001
EDVI (mL/m^2^)	45 ± 7.9	39 ± 8.7	0.005
ESVI (mL/m^2^)	14 ± 4.4	11 ± 3.7	<0.001
BW (${}^{\circ }$)	37.6 ± 7.3	36.7 ± 9.8	0.68
SD (${}^{\circ }$)	12.6 ± 2.8	12.2 ± 3.8	0.57

Data are shown as mean (SD) or median (IQR) for continuous variables, or as percentages for categorical variables.

BMI, body mass index; BSA, body surface area; 4DM, Corridor 4-dimensional model; EF, ejection fraction; EDV, end-diastolic volume; EDVI, end-diastolic volume index; ESV, end-systolic volume; ESVI, end-systolic volume index; BW, bandwidth; SD, standard deviation; E, entropy; QGS, quantitative gated single-photon emission computed tomography; ECTb, Emory Cardiac Toolbox.

## Discussion

In this study, we established preliminary clinical reference ranges for left ventricular function and synchronicity parameters using three software packages (4D-M, QGS, and ECTb) in patients with low-risk coronary artery disease who underwent stress GMPI in our center. In our population, a proportion of the subjects was derived from the cadre department, who regularly participate in physical activity. This may differ from the data collected by other centers. Therefore, we aimed to develop clinical reference ranges for these parameters based on the available data from our center. In our cohort, the proportion of women was relatively low (37/142, 26%); thus, the number of female groups was too small for a robust reference range calculation. We therefore acknowledge that all the female subgroup reference values are suggestive. Moreover, these preliminary values require validation in larger, well-powered cohorts before clinical implementation. It is recognized that the values determined using QGS are less precise in patients with small left ventricular volumes (21, 22). Akincioglu et al. (20) excluded patients with a small heart (defined as ESV<20 mL) because the primary objective of their research was to assess the feasibility of diastolic function evaluation using gated MPI. Nakajima et al. (5) defined a “small heart” as an ESV ≤20 mL using QGS software, based on observations from the Japanese multicenter J-ACCESS study, finding that three-quarters of women with low coronary disease likelihood had small hearts. Due to the high prevalence of having a small heart in our study, especially in the female groups (men: *n* = 20, 19% vs. women: *n* = 27, 73%), we included all the subjects with a small heart.

### Left ventricular function parameters

We compared left ventricular function parameters obtained using three quantitative software packages. The comparative analysis revealed that 4DM yielded the highest EF and EDV values, followed by ECTb, with QGS producing the lowest measurements. EF was comparable between QGS and ECTb, which were lower than that for 4DM. EDV and EDVI were comparable for 4DM and ECTb, which were higher than those for QGS. ESV was comparable between 4DM, QGS, and ECTb. ESVI obtained using 4DM was lower than when using ECTb. A previous study also found that the interchangeable use of different software packages should be avoided to prevent the overestimation or underestimation of left ventricular volume parameters (23). It is necessary to establish software-specific reference ranges for these parameters.

In this study, echocardiography data were also included. In total, 128 subjects were measured using two-dimensional echocardiography. QGS, ECTb, and 4D-M overestimated EF compared with echocardiography. The EDV values derived using 4DM and ECTb were comparable to those measured using echocardiography, which was inconsistent with the findings of a previous study (24). However, this previous study included patients with severe heart failure, while in our population, all the subjects had normal echocardiographic results with preserved EF. In this study, EF, EDV, and ESV were measured using M-mode echocardiography, while these parameters were measured using the Simpson method and a two-dimensional method in a previous study (19). Volume calculations derived from linear measurements in M-mode echocardiography may be more accurate (25). Previous studies have also found that left ventricular volumes and EF obtained using GMPI are less precise in patients with small left ventricular volumes compared with echocardiography (22) and MRI (26). This may partially explain the discrepancy in left ventricular volumes and EF evaluated using GMPI and echocardiography. It has been recommended that the interchangeable use of various imaging techniques to measure left ventricular EF and volumes should be avoided (27).

In our cohort, the proportion of men was relatively high (105/142, 74%). The male individuals were younger than the female individuals (male mean age, 45 ± 16.7 years; female mean age, 58 ± 15.2 years). The men had higher EDV/ESV and lower EF values than the women, which was consistent with previous studies (12, 28). After adjustment for BSA, EDVI and ESVI remained significantly higher in the men than the women. The cohort was selected from a consecutive series of patients who underwent GMPI at our center. Considering potential selection bias in sex, propensity score matching was employed, resulting in 37 matched pairs with comparable ages and BMI. Postmatching analysis confirmed higher EDV/ESV and EDVI/ESVI and lower EF in the men.

In addition, the prevalence of having a small heart was higher among the female subjects, which was in line with previous studies (12, 29). The prevalence of having a small heart among the male subjects in our study was lower than that of other studies. This may be related to the relatively younger age of the male subjects in our center.

In this study, age-related differences in left ventricular function parameters (including EDV, ESV, EDVI, and ESVI) were observed among the men. EF differed significantly between the two subgroups among the women. Previous studies have conflicting findings on age-related differences in EF and left ventricular volume. Nakajima et al. (5) reported that left ventricular volumes decrease significantly as age increases in men but not in women. LVEF did not correlate with age in men. This was partially similar to our findings. Li et al. (12) found age-related discrepancies in women but not in men. De Bondt et al. (7) also only found within-gender differences in EF or volumetric parameters between the different age groups in women in the ≥65 years age group, who had significantly higher EF and lower EDV and ESV values. A possible reason for the different findings may be differences in the participants’ age ranges and age distribution patterns across the different age groups in the studies. In our study, the multiple linear regression analysis showed that age was an important variable for EF and left ventricular volumes.

In our study, we further grouped the population based on different physical activity levels, i.e., subjects who participated in regular physical activity vs. those who were sedentary. In total, 48% (68/142) of the subjects regularly participated in physical activity. Among the men, the subgroup of subjects who regularly participated in physical activity was younger than the sedentary subgroup. They also had greater EDV, ESV, EDVI, and ESVI values. Among the women, the subgroup of subjects who regularly participated in physical activity was younger and had a lower EF value than the sedentary subgroup. They also had a larger ESV value (*P* < 0.05). The multiple linear regression analysis showed that gender, age, and BMI were important variables for EDV and ESV. Age and gender were found to be significant variables for EDVI, ESVI, and EF. Regularly participating in physical activity was not an independent influencing factor on EF and left ventricular volumes in our population. A previous study found that athlete's heart (AH) refers to a clinical picture characterized by a slow heart rate and enlargement of the heart, which is the result of morphological and functional cardiac modifications due to long-term athletic training (30). Exercise intensity and maximum aerobic capacity were found to influence cardiac adaptation in young competitive athletes (31). Our study did not include any professional athletes. All the individuals were regularly engaged in leisure-time physical exercise and the level of physical activity was not further graded. This may explain why regularly participating in physical activity did not significantly influence EF and left ventricular volumes. The influence of physical activity level on reference values remains to be further studied in the future.

The clinical reference values for left ventricular functional parameters obtained using different software packages in men and women were also established. Our results were relatively close to a previous study in spite of the use of different devices and image reconstruction methods (12). The men had higher EDV values obtained using QGS in our study than in Li et al.'s study (EDV: 85 ± 18.9 mL vs. 75 ± 18 mL). This difference may be due to there being a higher proportion of young people in the male subgroup in our study (≤40 years: 42/105 vs. 29/89).

### Left ventricular synchronicity parameters

Left ventricular mechanical synchronicity parameters, measured using various software packages, were also acquired. Consistent with previous research (13), clinical values for left ventricular synchronicity indices are different when derived from different software packages. In this study, the left ventricular systolic synchronicity parameters obtained using ECTb yielded the highest values, followed by QGS, while 4DM showed the lowest values. Thus, it is necessary to establish the clinical reference value ranges for each software package.

Chen et al. first proposed a normal database based on subjects with less than a 5% likelihood of coronary artery disease who underwent a standard ^201^Tl/^99m^Tc-sestamibi dual isotope rest/exercise stress protocol (32). Subsequently, a few studies have established normal references for left ventricular synchrony parameters. A systematic review found that normal phase analysis values can be different among healthy individuals even when the same software is used (14). The clinical reference values obtained from different populations were different (Table 11). The majority of studies are performed in Western populations. Trimble et al. (34) did not exclude those who were in abnormal sinus rhythm from the control group. The normal values obtained in their study were higher than in other studies. A few studies have established normal values in Asian populations. Mukherjee et al. (6) established a normal database for PSD and PBW in an Indian population. In their study, significant differences between synchronicity parameters were noted between men and women in both stress and rest studies. However, in our study, the left ventricular synchronicity parameters did not depend on sex, which is in accordance with previous studies (32, 36). Previous studies on sex differences in left ventricular synchronicity parameters have shown inconsistent results. A systematic review concluded that left ventricular synchronicity parameter values did not indicate relevant gender-based discrepancies after analyzing 13 published studies (14). A potential reason for the discrepancies among these studies may be significant heterogeneity in the enrolled patients regarding sample size, equipment, and age and gender distribution. We established clinical references for left ventricular synchronicity parameters in all the subjects. Cardiovascular risk factors, such as diabetes, hypertension, and dyslipidemia, could potentially affect left ventricular synchronicity. Ozdemir et al. (37) found that diabetes and hypertension could alter phase analysis parameters. Malik et al. (38) also found that long-term type II diabetes mellitus may affect phase analysis in patients with normal myocardial perfusion and LVEF. These cardiovascular risk factors were included because the study cohort was derived from a retrospective population with low pretest probability of significant coronary artery disease in clinical practice, rather than a rigorously screened and recruited healthy volunteer cohort. Thus, the clinical reference values in our study may not be the “true” normal values, but do reflect the real world.

**Table 11:** Comparison of left ventricular synchronicity parameters with other studies.

Author	Modality	Definition of controls	Age (years)	Number	Population	Algorithm	Instrument	SD(°)	BW(°)
Chen et al. (32)	Stress	No history of CAD, normal ECG, and no coronary artery calcium.	Not given	90	American	Emory Cardiac Toolbox	Not given	Female:11.8 ± 5.2 Male: 14.2 ± 5.1	Female: 30.6 ± 9.6 Male: 38.7 ± 11.8
Trimble et al. (33)	Stress	Ejection fraction ≥50% on gated SPECT myocardial perfusion imaging, without evidence of perfusion defects, without clinical history of coronary artery disease, with QRS durations ≤120 ms, and in normal sinus rhythm.	62	50	White, Black, others	Emory Cardiac Toolbox	Not given	8.6 ± 2.9	27.9 ± 8.9
Trimble et al. (34)	–	No history of cardiac disease, ejection fraction of greater than 50%, a less than 5% likelihood of coronary artery disease, and no evidence of LBBB or RBBB on surface electrocardiograms.	55	157	American	Emory Cardiac Toolbox	Not given	15.7 ± 11.8	42.0 ± 28.4
Atchley et al. (35)	stress	No history of CAD, normal myocardial perfusion imaging, and EF >55%.	61.2	75	European	Emory Cardiac Toolbox	Gamma camera	8.8 ± 3.1	28.7 ± 9.3
Mukherjee et al. (6)	Stress	Low pretest likelihood of coronary artery disease, no known history of cardiac disease, normal sinus rhythm on electrocardiogram (ECG), and QRS duration <120 ms.	52 ± 11.7	120	Indian	Emory Cardiac Toolbox	Hawkeye Infinia, General Electric Medical Systems, Waukesha, WI, USA	Male: 14.3 ± 4.7 Female: 11 ± 4	Male: 40.1 ± 11.9 Female: 34.7 ± 12.6
This study	Stress	Low-pretest likelihood of coronary artery disease, no known history of cardiac disease (e.g., CAD, cardiomyopathy, heart valve disease, and severe arrhythmia), normal perfusion at stress GMPI, normal ECG at rest, and LVEF >50%.	45 ± 17	142	Chinese	Emory Cardiac Toolbox	Optima NM/CT 640, GE Medical Systems, USA	Male: 13 ± 3.1 Female: 12 ± 3.8	Male: 38 ± 6.8 Female: 37 ± 9.8

CAD, coronary artery disease; ECG, electrocardiogram; SPECT, single-photon emission computed tomography; LBBB, left bundle branch block; RBBB, right bundle branch block; LVEF, left ventricular ejection fraction; GMPI, gated myocardial perfusion imaging; PSD, phase standard deviation; PBW, phase bandwidth.

Mukherjee et al. (6) compared rest- and stress-derived synchronicity indices. They found that low-dose stress images had significantly higher synchronicity indices compared to high-dose rest images, which is well-known in the assessment of cardiac mechanical dyssynchrony (39). In our study, only stress-derived synchronicity indices were obtained.

### Limitations

There were some limitations in our study. First, this was a retrospective single-center study. In our population, a proportion of the subjects were derived from the cadre department, who regularly participate in physical activity. Their health awareness and controlled cardiovascular risk factors are likely to be superior to those of the general population. This may differ from the data collected by other centers. The population was further grouped based on different physical activity levels, i.e., individuals who participated in regular physical activity vs. those who were sedentary. This could be an important factor that influences reference values. However, the multiple linear regression analysis showed that regularly participating in physical activity was not an independent influencing factor in our population. Nonetheless, caution should be exercised when extrapolating the results of this study to other populations. Clinical reference values were established in this study. Further validation of the reference values in population-based cohorts in future research will help clarify their general applicability. Second, the sample size was limited. As the proportion of female subjects was relatively low (37/142, 26%), we performed propensity score matching based on age and BMI to mitigate potential confounding effects. The postmatching comparison showed balanced baseline characteristics between the sexes, which enhanced the comparability of the derived reference values. However, the small proportion of female subjects may undermine the representativeness and statistical power of the reference values for left ventricular function and synchronicity parameters established in the female subgroups, which means these reference values are somewhat suggestive. Future studies should include a larger sample of women to establish clinical references. Third, this study included a population with low pretest likelihood of coronary artery disease who had a certain prevalence of diabetes (*n* = 14, 10%), hypertension (*n* = 44, 31%), and hyperlipidemia (*n* = 48, 34%). These cardiovascular risk factors in the cohort may have confounded the ventricular function and synchronicity values. Thus, the data may not be the “true” normal values, but do reflect the real world. Fourth, only stress gated myocardial images were obtained. Clinical references for rest GMPI were not established. Fifth, modern cardiac imaging guidelines recommend CT-based attenuation correction when available. Due to the retrospective study design, CT attenuation correction was not utilized in this study. A portion of the raw imaging data was acquired by a study protocol that did not include CT attenuation correction. *Post-hoc* correction was not feasible with the available reconstruction software. Future studies should incorporate CT attenuation correction when available.

## Conclusion

In this study, we established clinical reference values for left ventricular function and synchronicity parameters evaluated by GMPI using three quantitative software, namely Corridor 4D-M, QGS, and ECTb. Compared to the women, EDV, ESV, EDVI, and ESVI in the men were higher, while EF and the prevalence of having a small heart were lower. There were no significant differences in left ventricular synchronicity parameters between the men and women.

## Data Availability

The raw data supporting the conclusions of this article will be made available by the authors, without undue reservation.
